# Population pharmacokinetics study on nebulized and intravenous administration of polymyxin B in patients with pneumonia caused by multidrug-resistant gram-negative bacteria

**DOI:** 10.1128/aac.00044-25

**Published:** 2025-04-16

**Authors:** Xueyong Li, Lili Zhou, Danjie Wang, Qiong Wu, Xuanxi Huang, Hui Zhang, Wenwei Wu, Maobai Liu, Xuemei Wu, Hongqiang Qiu, Yu Cheng

**Affiliations:** 1 Department of Pharmacy, Fujian Medical University Union Hospital 731366, Fuzhou, Fujian, China; 2 Department of Critical Care Medicine, Fujian Medical University Union Hospital 117890, Fuzhou, Fujian, China; Providence Portland Medical Center, Portland, Oregon, USA

**Keywords:** polymyxin B, population pharmacokinetic, nebulized, multidrug-resistant, pneumonia

## Abstract

Polymyxin B (PMB) remains a last-line therapeutic agent for multidrug-resistant gram-negative bacteria (MDR-GNB) infections. However, reliable pharmacokinetic (PK) data to guide nebulized PMB dosing regimens in critically ill patients are limited. This study aimed to establish a population pharmacokinetic (PopPK) model for PMB in both epithelial lining fluid (ELF) and plasma of critically ill patients with MDR-GNB pneumonia and to optimize dosing regimens. A prospective PK study was conducted in 76 adult patients receiving nebulized PMB either as monotherapy or in combination with intravenous administration. PK data were analyzed using non-linear mixed-effect modeling, with PMB concentration–time profiles described by a coupled model integrating separate two-compartment models for plasma and ELF. The final model identified albumin levels and age as significant covariates influencing PK variability. Monte Carlo simulations demonstrated that nebulization therapy either alone or combined with intravenous administration significantly enhances ELF concentration and the probability of target attainment. Additionally, *Pseudomonas aeruginosa* requires higher nebulized doses than *Klebsiella pneumoniae* and *Acinetobacter baumannii*. This study develops a PopPK model of PMB in ELF and plasma, providing critical insights to optimize PMB treatment strategies for patients with MDR-GNB pneumonia.

## INTRODUCTION

Pneumonia represents the most prevalent nosocomial infection in intensive care units, associated with high morbidity and both short- and long-term mortality. Globally, it persists as the leading infectious cause of death across all age groups (1, 2). In critically ill populations, pneumonia manifests as community-acquired pneumonia, hospital-acquired pneumonia (HAP), or ventilator-associated pneumonia (VAP). Over the past several decades, the escalating prevalence of antibiotic resistance, particularly in multidrug-resistant strains of *Pseudomonas aeruginosa*, *Klebsiella pneumoniae*, and *Acinetobacter baumannii*, has introduced significant therapeutic challenges in severe pneumonia management (2).

Optimal pneumonia treatment requires the rapid initiation of pathogen-targeted antibiotics, with clinical efficacy fundamentally dependent on achieving adequate unbound drug concentrations at pulmonary infection sites (3). Multiple factors influence this process, including the extent of lung tissue penetration, the impact of pulmonary surfactants on antibiotic inactivation, plasma protein-binding capacity, and systemic clearance rates (4, 5). Insufficient consideration of these pharmacokinetic (PK) determinants may underlie the failure of conventional antibiotic regimens in HAP/VAP management (6, 7). Epithelial lining fluid (ELF) and alveolar macrophages are recognized as critical sites of infection for common extracellular and intracellular respiratory pathogens, respectively (4, 8). By measuring the concentration levels of antibiotics in ELF, their penetration into lung tissue can be characterized (4, 8). Therefore, elucidating the relationship between plasma and lung tissue antibiotic PK is essential to ensure that treatments for HAP and VAP achieve clinically sufficient drug exposure at the site of infection.

Polymyxin B (PMB) ranks among the earliest antibiotics that continue to be utilized in clinical practice. Discovered in the 1940s and approved in the 1950s, PMB has never undergone contemporary drug development procedures (9, 10). Although intravenous (IV) PMB utilization declined in the 1970s due to nephrotoxicity and neurotoxicity concerns, its clinical importance has resurged alongside escalating resistance to alternative antibiotics (9, 10). Consequently, PMB remains a critical last-line treatment for multidrug-resistant gram-negative bacterial (MDR-GNB) infections (11).

Polymyxin exhibits rapid bactericidal activity against susceptible MDR-GNB pathogens, including *Pseudomonas aeruginosa*, *Acinetobacter baumannii*, and *Klebsiella pneumoniae* (12). Concentrations exceeding the minimum inhibitory concentration (MIC) can lead to extremely rapid initial killing, with a significant reduction in colony-forming units per milliliter observed within 5 min of exposure (13). Preclinical evidence identifies the area under the concentration–time curve to MIC ratio of the free drug (*f*AUC/MIC) as the primary PK/pharmacodynamic (PD) index predictive of PMB efficacy (14–16). While IV PMB remains the standard clinical approach (11), achieving adequate *f*AUC/MIC in lung tissue is challenging due to variable pulmonary penetration (16–18). This limitation has driven interest in nebulized PMB as a strategy to enhance drug exposure at the infection site. Previous studies have demonstrated that adjunctive nebulized PMB therapy may augment lung tissue concentrations, potentially improving clinical responses and microbial eradication rates (19–22). However, existing studies are limited by small sample sizes, heterogeneous patient populations, and a lack of robust PK data to guide optimal dosing regimens.

Although population pharmacokinetics (PopPK) of systemic PMB administration have been described (9, 23), its pulmonary PK/PD characteristics have not yet been fully elucidated. More importantly, the ability of PMB to be effectively atomized into the lungs and achieve sufficient drug exposure at the site of infection has not been thoroughly investigated. Currently, there is a paucity of reliable PK data for nebulized PMB to inform dosage selection in nebulization therapy. Therefore, the primary objective of this study is to develop a PopPK model in the ELF and plasma of patients with MDR-GNB pneumonia infections and to utilize Monte Carlo simulations to optimize appropriate nebulized and IV dosage regimens.

## MATERIALS AND METHODS

### Subjects

This was a single-center, prospective study conducted between January 2022 and October 2024 at Fujian Medical University Union Hospital. The inclusion criteria for the study were as follows: (i) patients aged 18 years or older; (ii) clinical, laboratory, and imaging findings suggestive of pneumonia; and (iii) administration of nebulized PMB either as monotherapy or concomitantly with IV PMB for microbiologically proven infections caused by MDR-GNB. Patients with severe bronchoconstriction, refractory hypoxemia, pregnancy, or those undergoing continuous renal replacement therapy were excluded.

The following data were collected at the onset of the study: age, sex, weight, admission diagnosis, serum urea, creatinine clearance (CrCL), Acute Physiology and Chronic Health Evaluation (APACHE) II score, Sequential Organ Failure Assessment (SOFA) score, total bilirubin (TBIL), albumin (ALB), aspartate aminotransferase (AST), alanine aminotransferase (ALT), gamma-glutamyl transferase (GGT), alkaline phosphatase (ALP), interleukin-6 (IL-6), C-reactive protein (CRP), presence of sepsis, duration of mechanical ventilation, receipt of immunosuppressive therapy, and MIC for isolated pathogens and PMB. The CrCL was calculated using the Cockcroft–Gault formula.

### Administration of PMB

IV PMB was administered according to the manufacturer’s instructions: a loading dose of 100–150 mg (1 mg = 1 million units) followed by maintenance doses of 40–75 mg every 12 h, with an infusion time of approximately 1 h. The decision to administer PMB and its dosing regimen was made by the medical team. Concurrently, selected patients received nebulized PMB at a dose of 25 mg dissolved in 5 mL of half-normal sterile water for injection every 12 h via vibration mesh nebulizer or jet nebulizer over 15 min. Eligible patients received nebulized PMB for 3 days for the treatment of pneumonia. During nebulization, ventilation was performed at a constant flow, targeting an inspiration-to-expiration ratio of 0.5. The respiratory rate was set at 18 breaths/min; the tidal volume was set at 8 mL/kg; and the positive end-expiratory pressure was set at 6 cmH_2_O.

### Sampling procedures

For all patients, when the PMB concentration in the plasma and lungs reached a steady state (after at least four doses), one to six samples were collected into EDTA tubes at the following time points: 0 h before administration and 1, 2, 4, 6, 8, and 12 h after administration. The plasma and bronchoalveolar lavage (BAL) fluid samples for drug assays were centrifuged at 12,000 rpm for 5 min. The supernatant was then collected and stored at −80°C until analysis.

ELF is obtained through BAL. This procedure involves inserting and wedging a fiberoptic bronchoscope into a subsegment of the middle or lower lung lobe. Before performing BAL, the patient’s CT scan is reviewed to select the affected lung segment for lavage. The lavage is performed at the same location using a 0.9% saline solution. The aspirate from the first lavage is usually collected separately and discarded due to potential severe contamination with cells from the proximal airway. Subsequent recovered aspirates are combined, and their volume is measured and recorded. The remaining volume of BAL fluid is immediately centrifuged to separate the supernatant and the cell pellet. Samples used for measuring urea and drug concentrations in the supernatant are collected and frozen until analysis. The concentration of PMB in ELF can be determined using urea as an endogenous biomarker (4, 8). Urea concentration in plasma and ELF is typically the same because urea is non-polar, has a low molecular weight, and can rapidly diffuse to establish equilibrium across the capillary-alveolar membrane. By measuring the urea concentration in both BAL fluid and plasma, the dilution factor can be determined, allowing for the calculation of the actual drug concentration in ELF (Eq.1):


(1) $$C_{ELF,\;PMB}=C_{BAL,\;PMB}\times \frac{C_{plasma,\;urea}\;}{C_{BAL,\;urea}}.$$

### Determination of PMB concentration in plasma and bronchoalveolar lavage fluid by liquid chromatography–tandem mass spectrometry

As polymyxin B1 and B2 structures, molecular weights, pharmacological activities, and PK properties were identical, their plasma concentrations were summed to obtain total PMB concentrations (24). Concentrations of PMB were determined by a validated liquid chromatography–tandem mass spectrometry assay with minor modifications (25). The calibration curves showed acceptable linearity, ranging from 0.156 to 10.0 mg/L and from 0.0156 to 1.00 mg/L in the plasma for polymyxin B1 and B2, respectively, and were 0.1 to 150.0 and 0.01 to 10.0 mg/L for polymyxin B1 and B2, respectively, in BAL fluid. For plasma, intra- and interday accuracies ranged from 80.6% to 114.9%, with a coefficient of variation (CV) of 2.6%–14.8%. For BAL fluid, accuracy ranged from 82.3% to 114.3%, with a CV of 3.4%–13.7%. All sample concentrations exceeded the lower limit of quantification (LLOQ).

### Urea assay in plasma and bronchoalveolar lavage fluid

Urea concentrations in plasma and BAL fluid were quantified using a standardized enzymatic urease-glutamate dehydrogenase (GLDH) assay. Urea is hydrolyzed by urease to produce ammonia, which reacts with α-ketoglutarate and NADH in the presence of GLDH, forming glutamate and NAD^+^ (26). The rate of NADH oxidation, measured as absorbance decay at 340 nm (Δ*A*/min), correlates with urea concentration. Assays were performed on semi-automated biochemical analyzers (Chemray 800; Rayto Life and Analytical Sciences Co., Ltd., Shenzhen, China) at 37°C (Supplemental Methods).

Urea nitrogen (mg/dL) was calculated by multiplying urea (mmol/L) by 2.8. Plasma samples were collected in EDTA tubes, and BAL fluid was centrifuged (3,000 × *g*, 10 min) to remove debris. All samples were stored at −80°C to prevent urea degradation. The assay demonstrated a linear range of 0.5–100.0 mg/dL for both plasma and BAL samples. Intra- and interday accuracies in plasma ranged from 95.2% to 105.4%, with a CV of 3.7%–10.3%. For BAL fluid, accuracy ranged from 91.3% to 106.9%, with a CV of 4.3%–13.2%. Samples below the LLOQ were excluded from the analysis, including two plasma samples and seven BAL samples.

### Population pharmacokinetic modeling methods

PopPK analyses were conducted using a non-linear mixed-effect modeling program, NONMEM, version 7.5 (Icon Development Solutions, USA). Model development utilized first-order conditional estimation to assess the interaction between patient variability and residual variability. One- or two-compartment structural models were evaluated for both ELF and plasma drug exposure to establish the basic model that best fitted the original data. An exponential error model was used to describe interindividual variability, while residual variability was tested using additive, proportional, and combined error models. Covariates were not included prior to defining the basic structural model. The objective function value (OFV), which measures model improvement, was calculated using the difference in the −2 log likelihood for nested models. We investigated the impact of demographic and clinical factors deemed physiologically plausible for affecting PMB PK, including age, sex, weight, serum urea, CrCL, APACHE II score, SOFA score, TBIL, ALB, AST, ALT, GGT, ALP, IL-6, CRP, presence of sepsis, duration of mechanical ventilation, atomization types, and receipt of immunosuppressive therapy. Stepwise covariate modeling was conducted using a 5% forward inclusion and 1% backward elimination significance threshold. The OFV thresholds for forward inclusion and backward elimination were 3.84 and 6.63, respectively, corresponding to significance levels of 5% and 1%.

The final model was internally validated using goodness-of-fit (GOF) plots and visual predictive checks (VPCs) with 1,000 Monte Carlo simulations. Additionally, the bootstrap method was employed to generate 1,000 resampled data sets to analyze and evaluate the stability of the model.

### Monte Carlo simulation

Monte Carlo simulations were used to determine the probability of target attainment (PTA), aiding in the selection of the optimal dosage regimen. According to consensus guidelines, the *f*AUC/MIC is the most relevant PK/PD index associated with bacterial killing (11). In this study, we evaluated the PTA of *f*AUC/MIC across different dosage regimens in cases of pathogenic infection with varying MIC values to better understand the optimal treatment approach. The preclinical data on PMB are limited, and its *in vitro* activities are similar to those of colistin (11, 27). Consequently, some data on colistin were referenced when applying PK/PD indicators (11). In free plasma, the target *f*AUC/MIC for PMB is approximately 20, with the unbound fraction determined to be 0.42. Thus, an AUC/MIC of ≥50 is established as the therapeutic target, while considering nephrotoxicity, an AUC of >100 mg·h/L is set as the upper limit of the therapeutic range (11, 27). In ELF, the AUC/MIC targets associated with antibacterial activity against *Pseudomonas aeruginosa* (American Type Culture Collection [ATCC] 27853, PAO1, and FADDI-PA022) range from 1,326 to 1,506 (16); for *Acinetobacter baumannii* (ATCC 19606, 248–01-C.248, and N-16870.213), the targets range from 326 to 989; and for *Klebsiella pneumoniae* (ATCC BAA 2146, 17), the targets are between 525 and 527 (17).

This study utilized parameter estimates from the final model to conduct Monte Carlo simulations. A total of 1,000 simulations were performed on the original data set. Using the final model, we simulated dosage regimens for single IV administration, single nebulization, and combined IV and nebulization. The IV regimen consisted of maintenance doses of 50, 75, 100, 125, and 150 mg every 12 h (q12h) over 1 h, with a loading dose of twice the maintenance dose. The nebulization regimen included doses of 25, 50, 75, and 100 mg q12h, administered over 15 min/session. For each regimen, the PTA was calculated at 24 h and at steady state. Regimens were considered appropriate when PTA reached ≥90%.

## RESULTS

### Characteristics of the study population

**Fig 1:**
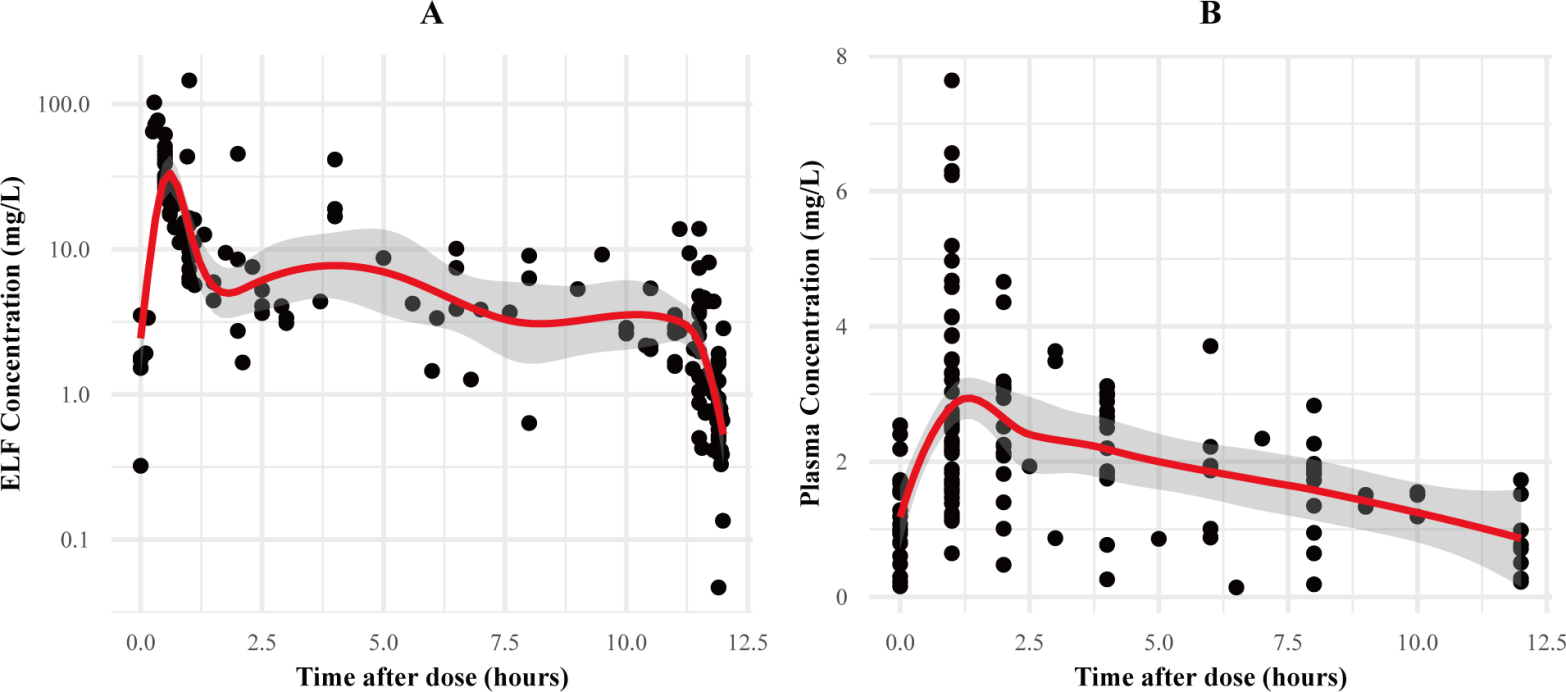
(**A**) ELF concentration–time profiles of PMB in 76 patients. (**B**) Plasma concentration–time profiles of PMB in 65 patients.

A total of 76 patients were included, from whom 156 BAL fluid samples were obtained from all patients and 188 plasma samples were obtained from 65 patients. The relationship between the PMB concentration and the time after administration is illustrated in Fig. 1. Table 1 summarizes the characteristic data, including demographic information, laboratory results, and pathogen details. Most patients were male (77.6%) and elderly, with a median age of 66 years. Additionally, infections with *Acinetobacter baumannii*, *Klebsiella pneumoniae*, and *Pseudomonas aeruginosa* accounted for 19.7%, 15.8%, and 18.4% of cases, respectively, while 46.1% of the patients were infected with two or more types of bacteria.

**TABLE 1:** Clinical characteristics of patients

Characteristics	Value^*a*^
Sex (male/female)	59/17
Height (cm)	170.0 (164.75–170.0)
Weight (kg)	65.0 (54.38–70.0)
Age (years)	66.0 (55.0–76.3)
APACHE II score	21.0 (13.0–27.0)
SOFA score	7.0 (4.0–9.3)
Total bilirubin (μmol/L)	14.4 (9.3–25.8)
Albumin (g/L)	32.1 (28.5–37.5)
Alanine aminotransferase (U/L)	23.0 (14.0–51.5)
Aspartate aminotransferase (U/L)	33.0 (25.0–53.0)
Urea nitrogen (mmol/L)	11.4 (6.8–17.8)
Creatinine clearance (mL/min)	55.7 (35.4–101.3)
Procalcitonin (ng/mL)	0.71 (0.27–2.89)
Interleukin-6 (pg/mL)	72.7 (31.3–184.8)
C-reactive protein (mg/L)	74.9 (28.3–112.8)
Pulmonary diseases, *n* (%)	13 (17.1)
Receipt of immunosuppressive therapy, *n* (%)	13 (17.1)
Duration of mechanical ventilation (days)	9.0 (4.5–12.0)
Pulmonary infection combined with sepsis, *n* (%)	42 (55.3)
AUC_24h_, _ELF_ (mg·h/L)	52.5 (35.3–102.8)
AUC_24h_, _plasma_ (mg·h/L)	35.0 (22.0–54.2)
Pathogenic bacteria, *n* (%)	
*Acinetobacter baumannii*	15 (19.7)
*Klebsiella pneumoniae*	12 (15.8)
*Pseudomonas aeruginosa*	14 (18.4)
Co-infection with two or more pathogenic bacteria	35 (46.1)
Daily intravenous dose (mg/kg)	1.82 (15.4–2.17)
Daily dose of nebulization (mg/kg)	0.77 (0.76–0.95)

^*a*^ Values are no. (%) or median (interquartile range).

### Population pharmacokinetic analysis for PMB in ELF and plasma

**Fig 2:**
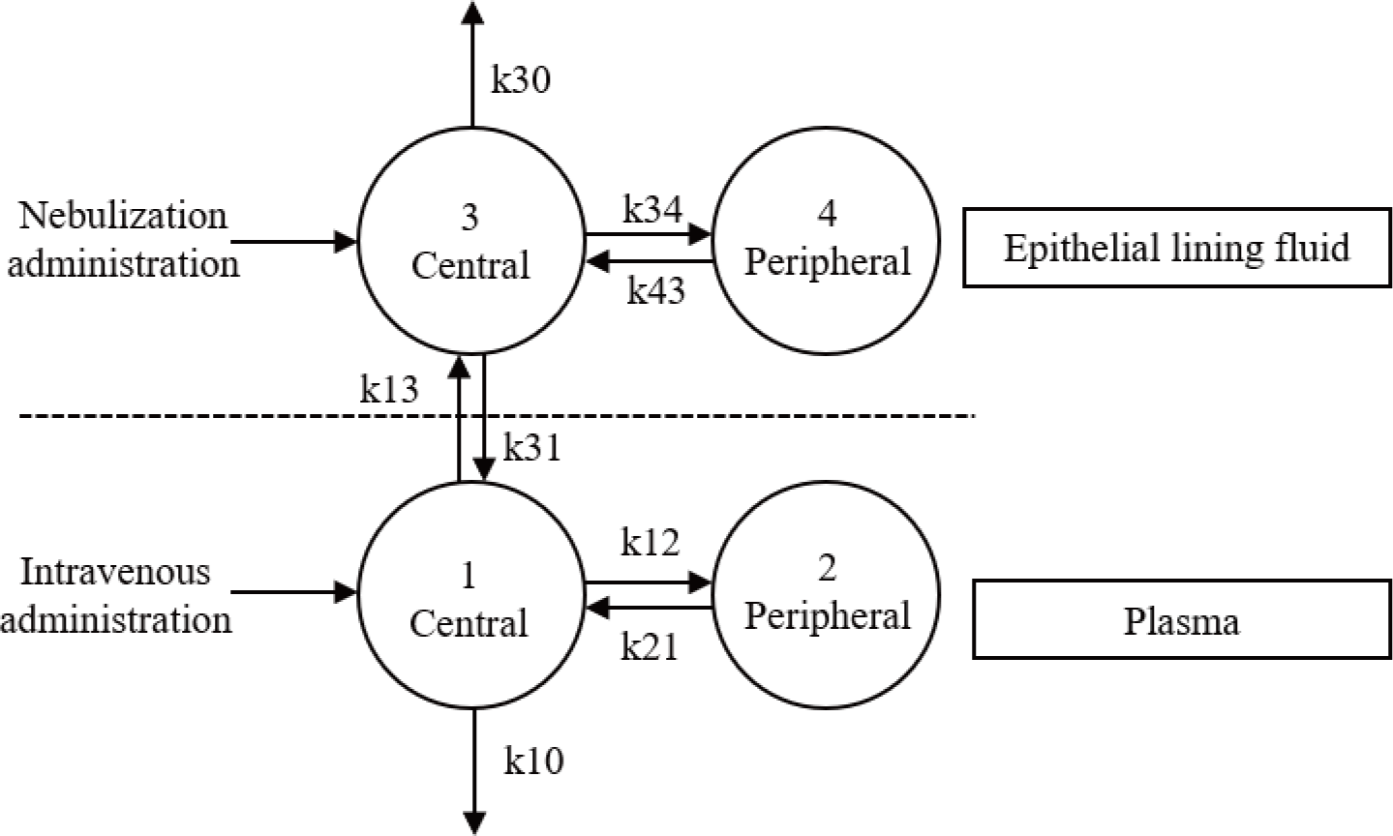
The final population pharmacokinetic model for intravenous and nebulized administration. *k*10 = CL1/*V*1, *k*12 = CL2/*V*1, *k*21 = CL1/*V*2, *k*13 = Q/*V*1, *k*31 = Q/*V*3, *k*30 = CL3/*V*3, *k*34 = CL4/*V*3, *k*43 = CL3*/V*4. CL, clearance rate; *k*, rate constant; *Q*, intercompartmental clearance between compartments 1 and 3; *V*, distribution volume.

The final model is presented in Fig. 2. A coupled model comprising two two-compartment models with linear elimination provided the best fit to adequately describe the collected data, based on the OFV and diagnostic plots. A proportional error model was described to evaluate residual variability. The development and selection process of the model are presented in Table S1. Overall, the forward inclusion and backward elimination results demonstrated that age and ALB significantly influenced the CL and *V* of the ELF central compartment. The final PopPK estimation parameters are presented in Table 2.

**TABLE 2:** PopPK parameter estimates for the final model and bootstrap analysis^*a*^

Parameter	Final model		Bootstrap	
	Estimate (shrinkage, %)	RSE %	Median	95% CI
CL1 (L/h)	1.39	6	1.39	1.28–1.49
*V*1 (L)	27.6	12	27.59	25.63–29.65
CL2 (L/h)	13.11	18	13.1	12.14–14.02
*V*2 (L)	17.1	11	17.11	15.80–18.38
CL3 (L/h)	0.186	21	0.186	0.174–0.201
*V*3 (L)	0.896	19	0.896	0.715–1.134
CL4 (L/h)	3.44	14	3.44	3.18–3.72
*V*4 (L)	4.35	10	4.33	4.02–4.67
*F*	0.557	21	0.556	0.51–0.60
*Q* (L/h)	0.03	10	0.03	0.027–0.032
AGE on *V*3	1.28	23	1.28	1.19–1.37
ALB on *CL*3	−0.893	17	−0.894	−1.06 to –0.73
Interindividual variability				
ω^2^CL1	0.56 (22%)	24	0.76	0.70–0.81
ω^2^V1	0.31 (18%)	15	0.3	0.28–0.33
ω^2^CL3	0.45 (25%)	23	0.74	0.69–0.80
ω^2^V3	0.53 (14%)	28	0.52	0.49–0.57
Residual variability				
Proportional error	0.43	13	0.43	0.40–0.45

^*a*^ ALB, albumin; CI, confidence interval; CL1/CL3, central compartment clearance of plasma or ELF; CL2/CL4, intercompartmental clearance of plasma or ELF; *F*, bioavailability of nebulization; *V*1/*V*3, central compartment distribution volume of plasma or ELF; *V*2/*V*4, intercompartmental distribution volume of plasma or ELF; RSE, relative standard error; *ω*^2^, variance of interindividual variability.

### Model validation

**Fig 3:**
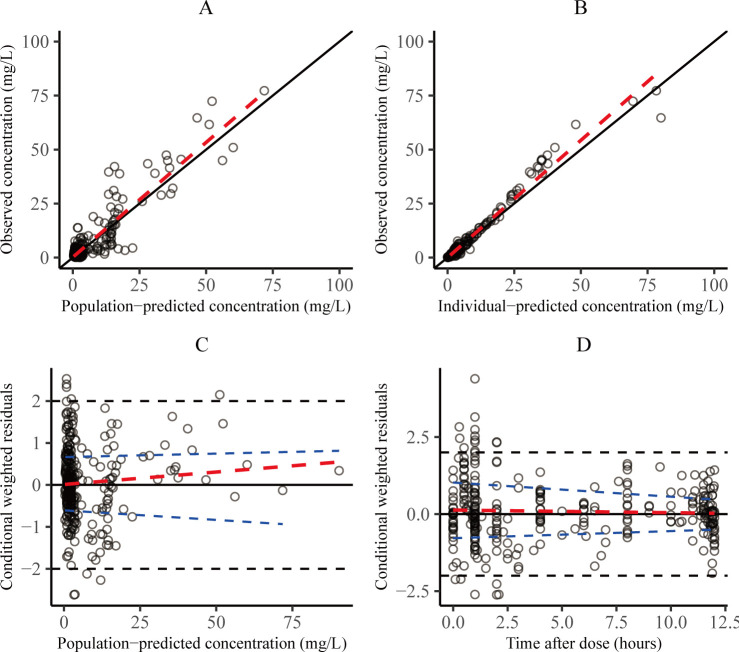
Goodness-of-fit plots of the final model. (**A**) Observed concentration vs. population-predicted concentration. (**B**) Observed concentration vs. individual-predicted concentration. (**C**) Conditional weighted residuals vs. population-predicted concentration. (**D**) Conditional weighted residuals vs. time after dose.

The GOF plots for the final model are presented in Fig. 3. No structural deviation was observed between population-predicted concentration (PRED) or individual-predicted concentration and observed concentration plots. The relationship between the conditional weighted residual of the final model and PRED was found to display a random distribution around zero, with the majority of residuals falling within the acceptable range of −2 to 2. Bootstrap analysis revealed that the parameter estimates of the final model were within the 95% CI, and the deviation of the parameter estimates was less than ±10% (Table 2), indicating that the final model has good stability. The VPCs of the 90% prediction interval for the final PopPK model demonstrate that the model performance is adequate, as presented in Fig. S1.

### Monte Carlo simulations

**Fig 4:**
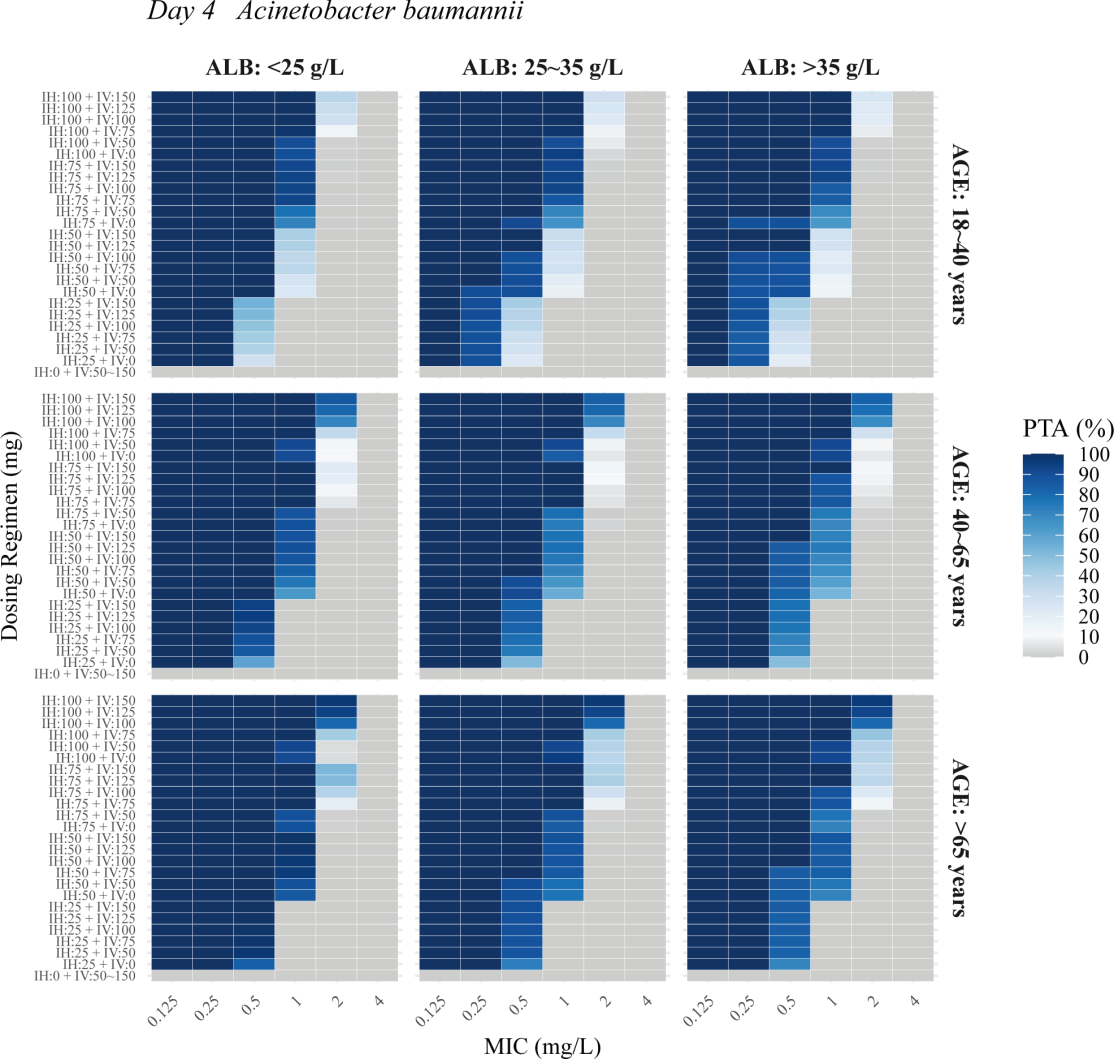
Probability of target attainment (PTA) in ELF at steady state (day 4) for *Acinetobacter baumannii* across various ages and albumin (ALB) levels in the final PopPK model. IH, nebulization maintenance dose; IV, intravenous maintenance dose.

**Fig 5:**
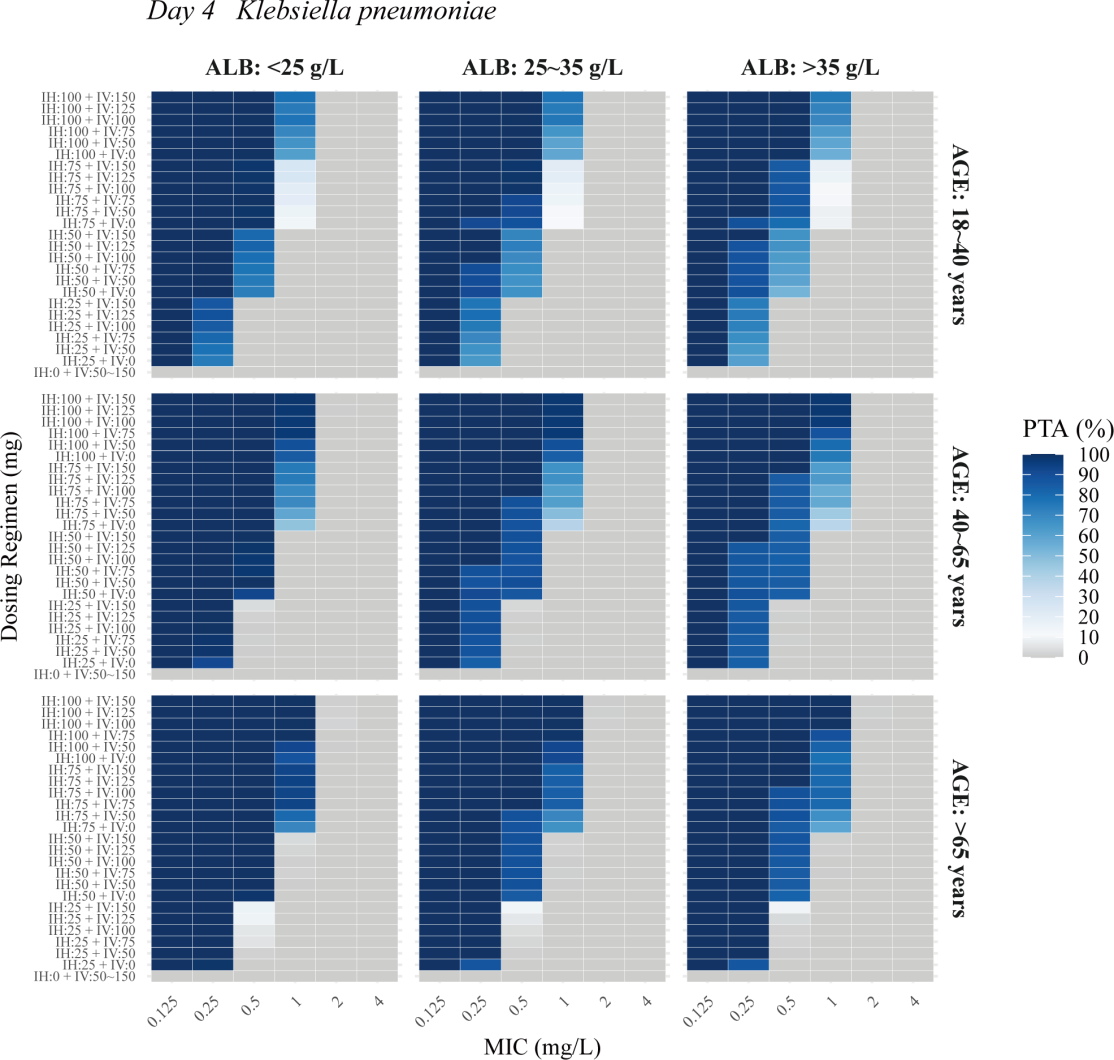
Probability of target attainment (PTA) in ELF at steady state (day 4) for *Klebsiella pneumoniae* across various ages and albumin (ALB) levels in the final PopPK model. IH, nebulization maintenance dose; IV, intravenous maintenance dose.

**Fig 6:**
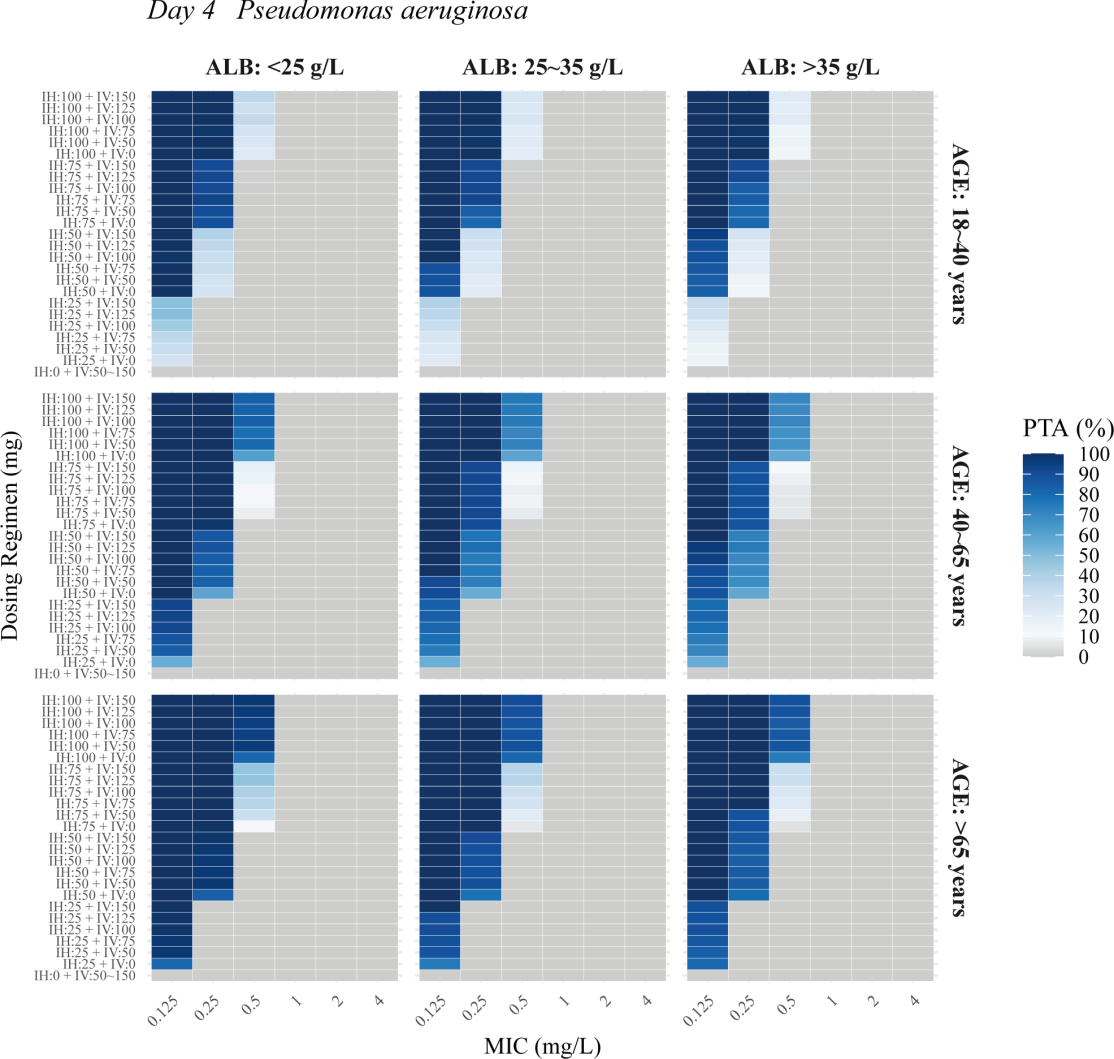
Probability of target attainment (PTA) in ELF at steady state (day 4) for *Pseudomonas aeruginosa* across various ages and albumin (ALB) levels in the final PopPK model. IH, nebulization maintenance dose; IV, intravenous maintenance dose.

The PTA analysis for nebulized and IV PMB was conducted across varying ALB levels (<25, 25–35L, and >35 g/L), age groups (18–40, 40–65, and >65 years), and MIC values for *Acinetobacter baumannii*, *Klebsiella pneumoniae*, and *Pseudomonas aeruginosa*. Results showed that higher age and lower ALB levels reduced the required dosage. Figure 4 to 6 illustrate the PTA in ELF at steady state for combined IV and nebulization therapy. The following regimens achieved a PTA of ≥90% at steady state: for *Klebsiella pneumoniae* and *Acinetobacter baumannii* with MICs of ≤0.25, 0.5, and 1.0 mg/L, nebulized doses of at least 25, 50, and 75 mg q12h, respectively, combined with IV doses of 0–75 mg q12h; and for *Pseudomonas aeruginosa* with MICs of ≤0.125 and 0.25 mg/L, nebulized doses of at least 50 and 75 mg q12h, respectively, combined with IV doses of 0–75 mg q12h. IV administration of 50–150 mg alone failed to achieve the PK/PD target values for the three bacteria in ELF.

When calculating PTA based on the *f*AUC_plasma_/MIC target through IV administration (Fig. S2), the following regimen achieved PTA of ≥90%: for MICs of ≤0.5 and 1 mg/L, IV doses of 50 and 75 mg q12h, respectively. However, higher IV maintenance doses increased toxicity risk (AUC >100 mg·h/L): 25%–30% at 75 mg q12h and >70% at 100 mg q12h (Fig. S3).

## DISCUSSION

Clinical data on PMB remain limited, particularly concerning its application in nebulization therapy for patients with pneumonia. It is essential to evaluate the enrichment capability of PMB nebulization in ELF, as the optimal therapeutic effect relies on the antibiotic’s ability to effectively reach the site of infection and achieve sufficient concentrations (11, 19, 28, 29). For pathogens such as *Pseudomonas aeruginosa* that cause severe pneumonia, determining the drug concentration in ELF and calculating pharmacodynamic indicators such as AUC/MIC are the most effective methods for evaluating the exposure levels of these antimicrobial agents (16–18). This study developed PopPK models for ELF and plasma in the pneumonia population to characterize the pharmacokinetic properties of PMB. Furthermore, it optimized the dosage regimen of nebulization and IV administration in these patients. To our knowledge, this is the first study to develop a PopPK model integrating PMB nebulization and IV administration for both ELF and plasma, offering critical insights for optimizing combined nebulized and IV dosing regimens in patients with MDR-GNB pneumonia.

The coupled model, consisting of two two-compartment models with first-order elimination effectively described PMB PK data across compartments, consistent with prior preclinical observations (16). In the ELF compartment, the CL and V for the central and peripheral compartments were 0.186 L/h, 0.896 L, 3.44 L/h, and 4.35 L, respectively, indicating complex pulmonary distribution dynamics. Lin et al. proposed that the first compartment in ELF might represent a reservoir of PMB in the upper respiratory tract, from which the drug is bidirectionally transferred to the peripheral lung region and partially enters lung epithelial cells (16). Initial attempts to incorporate interindividual variability (IIV) into peripheral compartment parameters (e.g., CL4 and *V*4) significantly increased model complexity and resulted in parameter instability (39% standard error for ALB on CL3) without improving OFV or predictive performance. Therefore, IIV for peripheral compartment parameters was excluded to ensure parameter reliability and interpretability while maintaining the model’s predictive performance. In addition, the fraction of drug reaching the lungs (*F* value) in the ELF model was 0.557, indicating that a significant portion of the drug does not fully reach the lungs during pulmonary administration. Although we evaluated two nebulization methods (jet atomization and vibration atomization), no significant effects were identified in the covariate analysis. This may be attributed to the fact that drug delivery efficiency depends not only on the nebulization method but also on factors such as nebulizer placement, synchronization with inhalation, humidification status, ventilator settings, and the patient interface (30). Furthermore, the droplet size distribution generated by the nebulizer critically influences aerosol losses in the breathing circuit (30). Therefore, for both invasive and non-invasive ventilation, delivery efficiency should be carefully evaluated for specific nebulizer, circuit, and ventilator combinations under anticipated clinical conditions (30).

This study incorporated ALB as a covariate in the PopPK model. To our knowledge, a decrease in ALB levels may lead to an increase in the free concentration of antibiotics with high protein affinity, thereby enhancing their clearance rate. A study on polymyxin interactions with human serum albumin (HSA) revealed that polymyxins bind to the cleft between domains I and III of HSA through electrostatic interactions, demonstrating a strong binding affinity (Ka ≈ 1.0 × 10⁷ M⁻¹) (31). This effect would be more pronounced in cases of severe lung inflammation, where serum albumin levels in the ELF space are elevated (6). Furthermore, this effect may also be associated with the expression of the peptide transporter PEPT2 in lung epithelial cells (16, 32). Previous studies confirmed that PEPT2 mediated the uptake of polymyxin in human embryonic kidney 293 cells (33). Given the high expression of PEPT2 in the airway epithelium, it may significantly influence the efficacy of polymyxin treatment (32). However, due to the lack of research on protein-binding effects in ELF, this study analyzed only the total drug concentration in ELF, and the impact of unbound drugs remains undetermined. Future research should further investigate the role of protein binding in ELF. Additionally, this study is the first to identify age as a key factor affecting the volume of distribution. This may be related to the presence of multiple exogenous metabolic enzymes in the lungs, which can influence the PK and safety of inhaled drugs (34). Drug carriers rely on pulmonary enzymes to trigger drug release or biodegradation, and the activity of these enzymes can vary, depending on age, race, smoking status, diet, drug exposure, and history of lung diseases (34, 35). This finding highlights the necessity of age-stratified dosing strategies for geriatric populations.

In the plasma compartment of the PopPK model, the CL and *V* for the central and peripheral compartments were as follows: CL1: 1.39 L/h, *V*1: 27.6 L, CL2: 13.11 L/h, and *V*2: 17.1 L, respectively. The CL1 aligned with previous studies on the PMB two-compartment model (CL1: 1.19–2.63 L/h), while the parameters for *V*1, *V*2, and CL2 exhibited significant variability across studies (*V*1: 6.35–33.77 L, CL2: 1.98–13.83 L/h, and *V*2: 7.92–78.2 L) (36–39). Notably, CrCL was not identified as a covariate in this study. The issue of dosage adjustments based on renal function has been a contentious topic in PMB pharmacological research. Sandri et al. reported that the urinary recovery rate of PMB ranged from 0.98% to 17.4% (39), while another study found an average urine recovery rate as high as 23.56% (40). In current PopPK studies of PMB, only some have included CrCL as a significant covariate (36, 40–44). This suggests considerable interindividual variability in the renal clearance rate of PMB. Consistent with current consensus guidelines, this study does not recommend dose adjustments for patients with renal insufficiency suffering from pneumonia (11). Additionally, this study indicated that the exchange rate between ELF and the plasma compartment was only 0.03 L/h, demonstrating that PMB exhibits limited permeability between lung tissue and plasma, consistent with previous findings (16, 25). Consequently, relying solely on PK/PD targets in plasma may result in insufficient drug exposure for effective pulmonary treatment.

Monte Carlo simulations demonstrated that *Pseudomonas aeruginosa* infection required higher nebulized doses compared to *Klebsiella pneumoniae* or *Acinetobacter baumannii* under the same conditions when treating MDR-GNB pneumonia with nebulization therapy. This is likely due to the significantly higher *f*AUC_ELF_/MIC targets for PMB against *Pseudomonas aeruginosa* strains (ATCC 27853, PAO1, and FADDI-PA022) in ELF ranging from 1,326 to 1,506 (16). A similar trend is observed with colistin, which also exhibits high *f*AUC_ELF_/MIC targets (684–1,050) for *Pseudomonas aeruginosa* (18). Such high *f*AUC/MIC levels are believed to be associated with the presence of multiple purulent emboli in obstructive bronchioles, which impede the delivery of nebulized PMB to infection sites (45). This phenomenon was observed in a piglet model infected with *Pseudomonas aeruginosa*, where the deposition of colistin aerosols was significantly impaired in cases of deep respiratory tract infections, potentially due to the presence of mucin and pulmonary surfactant (45). However, given the current empirical treatment protocols for PMB, high doses may increase the risk of pulmonary adverse reactions. Previous studies have shown that PMB can disrupt redox balance, mitochondrial beta-oxidation, and membrane lipid biosynthesis in human alveolar epithelial A549 cells (46, 47). Therefore, caution is advised when administering high-dose nebulized PMB.

This study demonstrates that IV infusion alone fails to achieve the PK/PD target in ELF due to the low exchange rate between plasma and ELF. While an IV loading dose enhances early plasma exposure of PMB, it has minimal impact on early ELF exposure. In contrast, nebulization alone or nebulization combined with IV administration significantly increases ELF concentrations and the PTA. Currently, the dosing regimen for PMB nebulization in clinical practice is predominantly empirical, typically involving doses of 25 or 50 mg administered twice daily (19–21). In a PK analysis of 105 patients with carbapenem-resistant pneumonia, adjunctive inhalation of PMB would be beneficial for patients unable to achieve the target concentration through IV administration (19). Additionally, in a retrospective study on ventilator-associated pneumonia caused by extensively drug-resistant GNB, the bacterial clearance time in the inhalation group was significantly shorter than in the IV group (*P* = 0.002), with a trend toward a reduced risk of acute kidney injury (*P* = 0.025) (21). Despite the European Society of Clinical Microbiology and Infectious Diseases position paper advising against the clinical use of nebulized antibiotics due to insufficient evidence from current trials (48), the international consensus guidelines of PMB and the treatment guidelines for VAP suggest that the potential benefits outweigh the associated risks (11, 28).

This study has several limitations. First, variations in BAL techniques among different researchers, particularly in factors such as residence time, suction pressure, volume of injected fluid, and the number of collected BAL samples (4, 49), may have influenced the correction of ELF dilution and the apparent volume of ELF. Therefore, it is recommended that standardized BAL procedures be established and consistently applied to minimize technical errors. Accurate understanding of research methodologies and strict adherence to operating protocols are essential for reducing known sources of error. Second, changes in ELF volume and protein concentration in BAL are closely associated with the condition of patients with a history of smoking and/or interstitial lung disease (50). Finally, the PK/PD data in this study primarily rely on preclinical experiments, which may not fully reflect clinical treatment conditions. Consequently, further clinical studies on the pulmonary administration of PMB are warranted.

### Conclusion

This study represents the first report of a PopPK model for PMB in both ELF and plasma of pneumonia patients, offering valuable insights for optimizing PMB treatment strategies for MDR-GNB pulmonary infections. The study identified age and ALB levels as significant covariates influencing the PopPK model in ELF and plasma. These findings provide a reference for PMB administration via nebulization and intravenous routes in patients with pneumonia caused by MDR-GNB. Further research is warranted to explore the clinical application of PK/PD-based nebulized PMB therapy.

## Data Availability

All data sets analyzed in the present study are included in the article or Supplemental material.

## SUPPLEMENTAL MATERIAL

Supplemental material aac.00044-25-s0001.docx Table S1; Fig. S1 to S3.

Supplemental methods aac.00044-25-s0002.docx Text and table.

ASM does not own the copyrights to Supplemental Material that may be linked to, or accessed through, an article. The authors have granted ASM a non-exclusive, world-wide license to publish the Supplemental Material files. Please contact the corresponding author directly for reuse.

## References

[1] Cillóniz C, Torres A, Niederman MS. 2021. Management of pneumonia in critically ill patients. BMJ 375:e065871. doi:10.1136/bmj-2021-065871 34872910

[2] Zaragoza R, Vidal-Cortés P, Aguilar G, Borges M, Diaz E, Ferrer R, Maseda E, Nieto M, Nuvials FX, Ramirez P, Rodriguez A, Soriano C, Veganzones J, Martín-Loeches I. 2020. Update of the treatment of nosocomial pneumonia in the ICU. Crit Care 24:383. doi:10.1186/s13054-020-03091-2 32600375 PMC7322703

[3] Gonzalez D, Schmidt S, Derendorf H. 2013. Importance of relating efficacy measures to unbound drug concentrations for anti-infective agents. Clin Microbiol Rev 26:274–288. doi:10.1128/CMR.00092-12 23554417 PMC3623378

[4] Kiem S, Schentag JJ. 2008. Interpretation of antibiotic concentration ratios measured in epithelial lining fluid. Antimicrob Agents Chemother 52:24–36. doi:10.1128/AAC.00133-06 17846133 PMC2223903

[5] Rodvold KA, George JM, Yoo L. 2011. Penetration of anti-infective agents into pulmonary epithelial lining fluid: focus on antibacterial agents. Clin Pharmacokinet 50:637–664. doi:10.2165/11594090-000000000-00000 21895037

[6] Rodvold KA, Hope WW, Boyd SE. 2017. Considerations for effect site pharmacokinetics to estimate drug exposure: concentrations of antibiotics in the lung. Curr Opin Pharmacol 36:114–123. doi:10.1016/j.coph.2017.09.019 29096171

[7] Ambrose PG, Bhavnani SM, Ellis-Grosse EJ, Drusano GL. 2010. Pharmacokinetic-pharmacodynamic considerations in the design of hospital-acquired or ventilator-associated bacterial pneumonia studies: look before you leap! Clin Infect Dis 51 Suppl 1:S103–10. doi:10.1086/653057 20597657

[8] Rennard SI, Basset G, Lecossier D, O’Donnell KM, Pinkston P, Martin PG, Crystal RG. 1986. Estimation of volume of epithelial lining fluid recovered by lavage using urea as marker of dilution. J Appl Physiol (1985) 60:532–538. doi:10.1152/jappl.1986.60.2.532 3512509

[9] Avedissian SN, Liu J, Rhodes NJ, Lee A, Pais GM, Hauser AR, Scheetz MH. 2019. A review of the clinical pharmacokinetics of polymyxin B. Antibiotics (Basel) 8:31. doi:10.3390/antibiotics8010031 30909507 PMC6466567

[10] Nang SC, Azad MAK, Velkov T, Zhou QT, Li J. 2021. Rescuing the last-line polymyxins: achievements and challenges. Pharmacol Rev 73:679–728. doi:10.1124/pharmrev.120.000020 33627412 PMC7911091

[11] Tsuji BT, Pogue JM, Zavascki AP, Paul M, Daikos GL, Forrest A, Giacobbe DR, Viscoli C, Giamarellou H, Karaiskos I, Kaye D, Mouton JW, Tam VH, Thamlikitkul V, Wunderink RG, Li J, Nation RL, Kaye KS. 2019. International Consensus Guidelines for the Optimal Use of the Polymyxins: Endorsed by the American College of Clinical Pharmacy (ACCP), European Society of Clinical Microbiology and Infectious Diseases (ESCMID), Infectious Diseases Society of America (IDSA), International Society for Anti-infective Pharmacology (ISAP), Society of Critical Care Medicine (SCCM), and Society of Infectious Diseases Pharmacists (SIDP). Pharmacotherapy 39:10–39. doi:10.1002/phar.2209 30710469 PMC7437259

[12] Zavascki AP, Goldani LZ, Li J, Nation RL. 2007. Polymyxin B for the treatment of multidrug-resistant pathogens: a critical review. J Antimicrob Chemother 60:1206–1215. doi:10.1093/jac/dkm357 17878146

[13] Bergen PJ, Li J, Nation RL, Turnidge JD, Coulthard K, Milne RW. 2008. Comparison of once-, twice- and thrice-daily dosing of colistin on antibacterial effect and emergence of resistance: studies with Pseudomonas aeruginosa in an in vitro pharmacodynamic model. J Antimicrob Chemother 61:636–642. doi:10.1093/jac/dkm511 18227094

[14] Landersdorfer CB, Wang J, Wirth V, Chen K, Kaye KS, Tsuji BT, Li J, Nation RL. 2018. Pharmacokinetics/pharmacodynamics of systemically administered polymyxin B against Klebsiella pneumoniae in mouse thigh and lung infection models. J Antimicrob Chemother 73:462–468. doi:10.1093/jac/dkx409 29149294 PMC5890666

[15] Cheah S-E, Wang J, Nguyen VTT, Turnidge JD, Li J, Nation RL. 2015. New pharmacokinetic/pharmacodynamic studies of systemically administered colistin against Pseudomonas aeruginosa and Acinetobacter baumannii in mouse thigh and lung infection models: smaller response in lung infection. J Antimicrob Chemother 70:3291–3297. doi:10.1093/jac/dkv267 26318190

[16] Lin Y-W, Zhou Q, Onufrak NJ, Wirth V, Chen K, Wang J, Forrest A, Chan H-K, Li J. 2017. Aerosolized polymyxin B for treatment of respiratory tract infections: determination of pharmacokinetic-pharmacodynamic indices for aerosolized polymyxin B against Pseudomonas aeruginosa in a mouse lung infection model. Antimicrob Agents Chemother 61:e00211-17. doi:10.1128/AAC.00211-17 28559256 PMC5527630

[17] Lin Y-W, Zhou QT, Han M-L, Chen K, Onufrak NJ, Wang J, Turnidge JD, Howden BP, Forrest A, Chan H-K, Li J. 2018. Elucidating the Pharmacokinetics/Pharmacodynamics of aerosolized colistin against multidrug-resistant Acinetobacter baumannii and Klebsiella pneumoniae in a mouse lung infection model. Antimicrob Agents Chemother 62:e01790-17. doi:10.1128/AAC.01790-17 29229637 PMC5786800

[18] Lin Y-W, Zhou QT, Cheah S-E, Zhao J, Chen K, Wang J, Chan H-K, Li J. 2017. Pharmacokinetics/pharmacodynamics of pulmonary delivery of colistin against Pseudomonas aeruginosa in a mouse lung infection model. Antimicrob Agents Chemother 61:e02025-16. doi:10.1128/AAC.02025-16 28031207 PMC5328518

[19] Tang T, Li Y, Xu P, Zhong Y, Yang M, Ma W, Xiang D, Zhang B, Zhou Y. 2023. Optimization of polymyxin B regimens for the treatment of carbapenem-resistant organism nosocomial pneumonia: a real-world prospective study. Crit Care 27:164. doi:10.1186/s13054-023-04448-z 37106370 PMC10142183

[20] Ding P, Li H, Nan Y, Liu C, Wang G, Cai H, Yu W. 2024. Outcome of intravenous and inhaled polymyxin B treatment in patients with multidrug-resistant gram-negative bacterial pneumonia. Int J Antimicrob Agents 64:107293. doi:10.1016/j.ijantimicag.2024.107293 39094752

[21] Shi R, Fu Y, Gan Y, Wu D, Zhou S, Huang M. 2023. Use of polymyxin B with different administration methods in the critically ill patients with ventilation associated pneumonia: a single-center experience. Front Pharmacol 14:1222044. doi:10.3389/fphar.2023.1222044 37719858 PMC10502420

[22] Pereira GH, Muller PR, Levin AS. 2007. Salvage treatment of pneumonia and initial treatment of tracheobronchitis caused by multidrug-resistant Gram-negative bacilli with inhaled polymyxin B. Diagn Microbiol Infect Dis 58:235–240. doi:10.1016/j.diagmicrobio.2007.01.008 17350201

[23] Hanafin PO, Nation RL, Scheetz MH, Zavascki AP, Sandri AM, Kwa AL, Cherng BPZ, Kubin CJ, Yin MT, Wang J, Li J, Kaye KS, Rao GG. 2021. Assessing the predictive performance of population pharmacokinetic models for intravenous polymyxin B in critically ill patients. CPT Pharmacometrics Syst Pharmacol 10:1525–1537. doi:10.1002/psp4.12720 34811968 PMC8674003

[24] He J, Ledesma KR, Lam W-Y, Figueroa DA, Lim T-P, Chow D-L, Tam VH. 2010. Variability of polymyxin B major components in commercial formulations. Int J Antimicrob Agents 35:308–310. doi:10.1016/j.ijantimicag.2009.11.005 20045285

[25] Zhang B, Li X, Chen Y, Chen B, Cheng Y, Lin H, Que W, Liu M, Zhou L, Zhang H, Qiu H, Wu C. 2023. Determination of polymyxin B in human plasma and epithelial lining fluid using LC-MS/MS and its clinical application in therapeutic drug monitoring. J Pharm Biomed Anal 227:115291. doi:10.1016/j.jpba.2023.115291 36822067

[26] TALKE H, SCHUBERT GE. 1965. Enzymatic urea determination in the blood and serum in the warburg optical test. Klin Wochenschr 43:174–175. doi:10.1007/BF01484513 14258517

[27] Sader HS, Rhomberg PR, Farrell DJ, Jones RN. 2015. Differences in potency and categorical agreement between colistin and polymyxin B when testing 15,377 clinical strains collected worldwide. Diagn Microbiol Infect Dis 83:379–381. doi:10.1016/j.diagmicrobio.2015.08.013 26415906

[28] Kalil AC, Metersky ML, Klompas M, Muscedere J, Sweeney DA, Palmer LB, Napolitano LM, O’Grady NP, Bartlett JG, Carratalà J, El Solh AA, Ewig S, Fey PD, File TM Jr, Restrepo MI, Roberts JA, Waterer GW, Cruse P, Knight SL, Brozek JL. 2016. Management of adults with hospital-acquired and ventilator-associated pneumonia: 2016 clinical practice guidelines by the infectious diseases society of America and the American thoracic society. Clin Infect Dis 63:e61–e111. doi:10.1093/cid/ciw353 27418577 PMC4981759

[29] He J, Abdelraouf K, Ledesma KR, Chow D-L, Tam VH. 2013. Pharmacokinetics and efficacy of liposomal polymyxin B in a murine pneumonia model. Int J Antimicrob Agents 42:559–564. doi:10.1016/j.ijantimicag.2013.07.009 24016799 PMC3849129

[30] Martin AR, Finlay WH. 2015. Nebulizers for drug delivery to the lungs. Expert Opin Drug Deliv 12:889–900. doi:10.1517/17425247.2015.995087 25534396

[31] Poursoleiman A, Karimi-Jafari MH, Zolmajd-Haghighi Z, Bagheri M, Haertlé T, Behbehani GR, Ghasemi A, Stroylova YY, Muronetz VI, Saboury AA. 2019. Polymyxins interaction to the human serum albumin: a thermodynamic and computational study. Spectrochim Acta A Mol Biomol Spectrosc 217:155–163. doi:10.1016/j.saa.2019.03.077 30933779

[32] Bosquillon C. 2010. Drug transporters in the lung--do they play a role in the biopharmaceutics of inhaled drugs? J Pharm Sci 99:2240–2255. doi:10.1002/jps.21995 19950388

[33] Lu X, Chan T, Xu C, Zhu L, Zhou QT, Roberts KD, Chan H-K, Li J, Zhou F. 2016. Human oligopeptide transporter 2 (PEPT2) mediates cellular uptake of polymyxins. J Antimicrob Chemother 71:403–412. doi:10.1093/jac/dkv340 26494147 PMC4710213

[34] Enlo-Scott Z, Bäckström E, Mudway I, Forbes B. 2021. Drug metabolism in the lungs: opportunities for optimising inhaled medicines. Expert Opin Drug Metab Toxicol 17:611–625. doi:10.1080/17425255.2021.1908262 33759677

[35] Miller MR. 2010. Structural and physiological age-associated changes in aging lungs. Semin Respir Crit Care Med 31:521–527. doi:10.1055/s-0030-1265893 20941653

[36] Hanafin PO, Kwa A, Zavascki AP, Sandri AM, Scheetz MH, Kubin CJ, Shah J, Cherng BPZ, Yin MT, Wang J, Wang L, Calfee DP, Bolon M, Pogue JM, Purcell AW, Nation RL, Li J, Kaye KS, Rao GG. 2023. A population pharmacokinetic model of polymyxin B based on prospective clinical data to inform dosing in hospitalized patients. Clin Microbiol Infect 29:1174–1181. doi:10.1016/j.cmi.2023.05.018 37217076

[37] Wang P, Zhang Q, Zhu Z, Pei H, Feng M, Sun T, Yang J, Zhang X. 2021. Comparing the population pharmacokinetics of and acute kidney injury due to polymyxin B in chinese patients with or without renal insufficiency. Antimicrob Agents Chemother 65:e01900-20. doi:10.1128/AAC.01900-20 33168613 PMC7848972

[38] Miglis C, Rhodes NJ, Avedissian SN, Kubin CJ, Yin MT, Nelson BC, Pai MP, Scheetz MH. 2018. Population pharmacokinetics of polymyxin B in acutely ill adult patients. Antimicrob Agents Chemother 62:e01475. doi:10.1128/AAC.01475-17 29311071 PMC5826159

[39] Sandri AM, Landersdorfer CB, Jacob J, Boniatti MM, Dalarosa MG, Falci DR, Behle TF, Bordinhão RC, Wang J, Forrest A, Nation RL, Li J, Zavascki AP. 2013. Population pharmacokinetics of intravenous polymyxin B in critically ill patients: implications for selection of dosage regimens. Clin Infect Dis 57:524–531. doi:10.1093/cid/cit334 23697744

[40] Yu X-B, Jiao Z, Zhang C-H, Dai Y, Zhou Z-Y, Han L, Wen X, Sheng C-C, Lin G-Y, Pan J-Y. 2021. Population pharmacokinetic and optimization of polymyxin B dosing in adult patients with various renal functions. Br J Clin Pharmacol 87:1869–1877. doi:10.1111/bcp.14576 33002196

[41] Avedissian SN, Miglis C, Kubin CJ, Rhodes NJ, Yin MT, Cremers S, Prickett M, Scheetz MH. 2018. Polymyxin B pharmacokinetics in adult cystic fibrosis patients. Pharmacotherapy 38:730–738. doi:10.1002/phar.2129 29800496

[42] Wang P, Zhang Q, Zhu Z, Feng M, Sun T, Yang J, Zhang X. 2020. Population pharmacokinetics and limited sampling strategy for therapeutic drug monitoring of polymyxin B in Chinese patients with multidrug-resistant Gram-negative bacterial infections. Front Pharmacol 11:829. doi:10.3389/fphar.2020.00829 32581795 PMC7289991

[43] Li Y, Deng Y, Zhu Z-Y, Liu Y-P, Xu P, Li X, Xie Y-L, Yao H-C, Yang L, Zhang B-K, Zhou Y-G. 2021. Population pharmacokinetics of polymyxin B and dosage optimization in renal transplant patients. Front Pharmacol 12:727170. doi:10.3389/fphar.2021.727170 34512352 PMC8424097

[44] Li X, Cheng Y, Chen B, Chen Y, Huang Y, Zhang B, Que W, Liu M, Zhang H, Qiu H. 2023. Population pharmacokinetics of polymyxin B in patients with liver dysfunction. Brit J Clinical Pharma 89:3561–3572. doi:10.1111/bcp.15855 37461291

[45] Lu Q, Girardi C, Zhang M, Bouhemad B, Louchahi K, Petitjean O, Wallet F, Becquemin M-H, Le Naour G, Marquette C-H, Rouby J-J. 2010. Nebulized and intravenous colistin in experimental pneumonia caused by Pseudomonas aeruginosa. Intensive Care Med 36:1147–1155. doi:10.1007/s00134-010-1879-4 20397007

[46] Ahmed MU, Azad MAK, Li M, Creek DJ, Han M, Zhou F, Chan K, Zhou QT, Velkov T, Li J. 2021. Polymyxin-induced metabolic perturbations in human lung epithelial cells. Antimicrob Agents Chemother 65:e0083521. doi:10.1128/AAC.00835-21 34228550 PMC8376244

[47] Ahmed MU, Velkov T, Lin Y-W, Yun B, Nowell CJ, Zhou F, Zhou QT, Chan K, Azad MAK, Li J. 2017. Potential toxicity of polymyxins in human lung epithelial cells. Antimicrob Agents Chemother 61:e02690-16. doi:10.1128/AAC.02690-16 28416543 PMC5444173

[48] Rello J, Solé-Lleonart C, Rouby J-J, Chastre J, Blot S, Poulakou G, Luyt C-E, Riera J, Palmer LB, Pereira JM, Felton T, Dhanani J, Bassetti M, Welte T, Roberts JA. 2017. Use of nebulized antimicrobials for the treatment of respiratory infections in invasively mechanically ventilated adults: a position paper from the European Society of Clinical Microbiology and Infectious Diseases. Clin Microbiol Infect 23:629–639. doi:10.1016/j.cmi.2017.04.011 28412382

[49] Baldwin DR, Honeybourne D, Wise R. 1992. Pulmonary disposition of antimicrobial agents: methodological considerations. Antimicrob Agents Chemother 36:1171–1175. doi:10.1128/AAC.36.6.1171 1416816 PMC190295

[50] Roberts CM, Cairns D, Bryant DH, Burke WM, Yeates M, Blake H, Penny R, Shelley L, Zaunders JJ, Breit SN. 1993. Changes in epithelial lining fluid albumin associated with smoking and interstitial lung disease. Eur Respir J 6:110–115. 8425580

