# Supplementary material for: Population pharmacokinetics study on nebulized and intravenous administration of polymyxin B in patients with pneumonia caused by multidrug-resistant gram-negative bacteria
Source: Antimicrob Agents Chemother. 2025 Apr 16;69(5):e00044-25. doi: 10.1128/aac.00044-25 (PMC12057357; doi:10.1128/aac.00044-25)
Supplement: Supplemental material — Table S1; Fig. S1 to S3. [file aac.00044-25-s0001.docx]

Table S1. Model selection and development.

| Model | Model description | OFV | ΔOFV | *P* value |
| --- | --- | --- | --- | --- |
|  | IV-1CMT+IH-1CMT | 796.439 | - | - |
|  | IV-1CMT+IH-2CMT | 788.108 |  |  |
|  | IV-2CMT+IH-1CMT | 796.574 |  |  |
|  | IV-2CMT+IH-2CMT | 783.066 | - | - |
| The choice of residual variability model | | | | |
| 1 | Addictive error model | 818.374 | - | - |
| 2 | Proportional error model | 783.046 | - | - |
| 3 | Combined error model | 783.066 | - | - |
| Forward inclusion | |  |  |  |
| 2 | Basic model | 783.046 | - | - |
| 4 | Add AGE on V3 in model 2 | 775.776 | -7.27 | < 0.05 |
| 5 | Add ALB on CL3 in model 4 | 769.964 | -5.812 | < 0.05 |
| Backward elimination | |  |  |  |
| 6 | Remove AGE on V3 in model 5 | 785.635 | 15.671 | < 0.001 |
| 7 | Remove ALB on CL3 in model 5 | 780.894 | 10.93 | < 0.001 |

Note: IV, intravenous administration; IH, nebulization; CMT, compartment; ALB, albumin; V3, central compartment distribution volume of ELF; CL3, central compartment clearance of ELF; OFV, objective function value; *P* value, a decrease in the OFV was referred to as the χ^2^ distributions to assess significance.


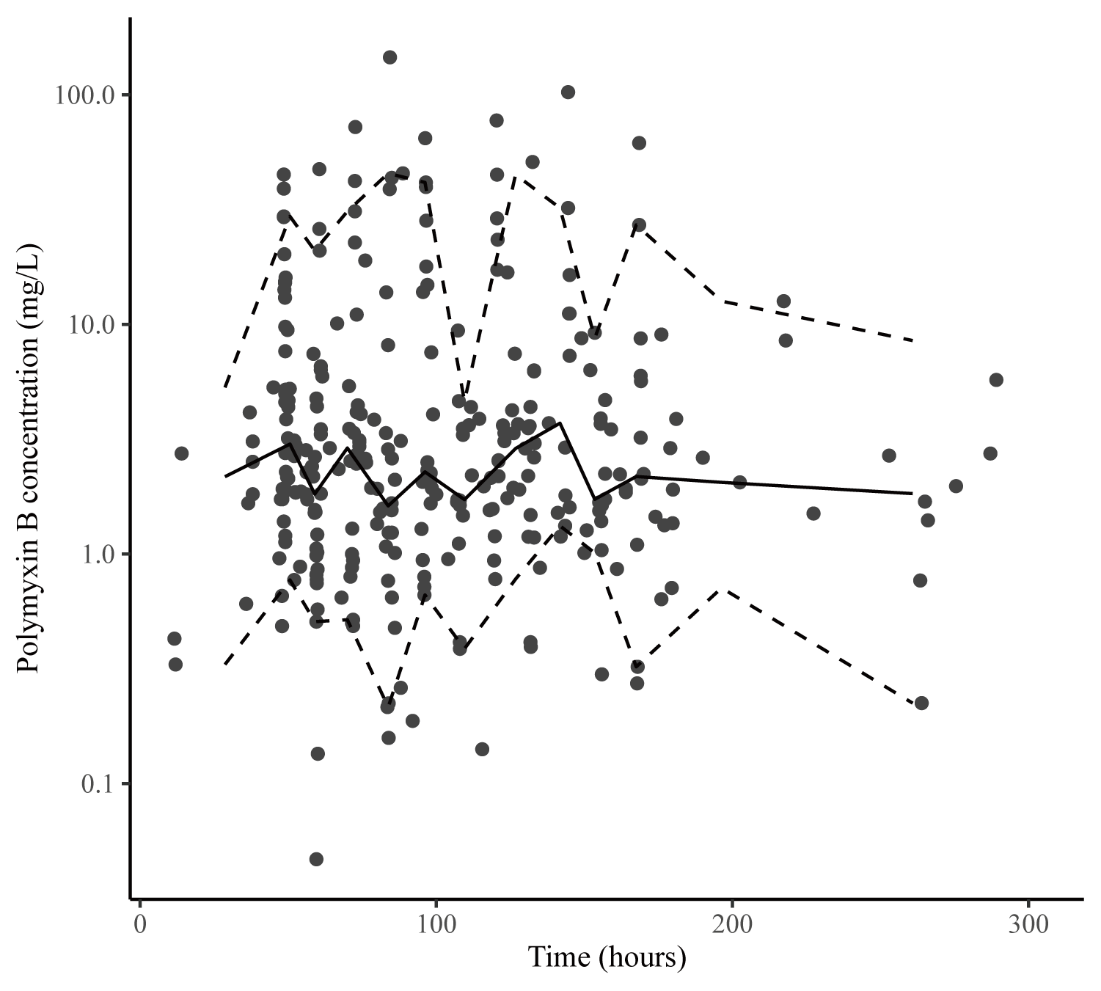


Figure S1. Visual predictive checks displaying observed and predicted concentrations over time based on the final polymyxin B population pharmacokinetic model. Filled circles represent observed concentrations; dashed lines indicate the 5th and 95th percentile predicted concentrations; solid lines show the median predicted concentrations.


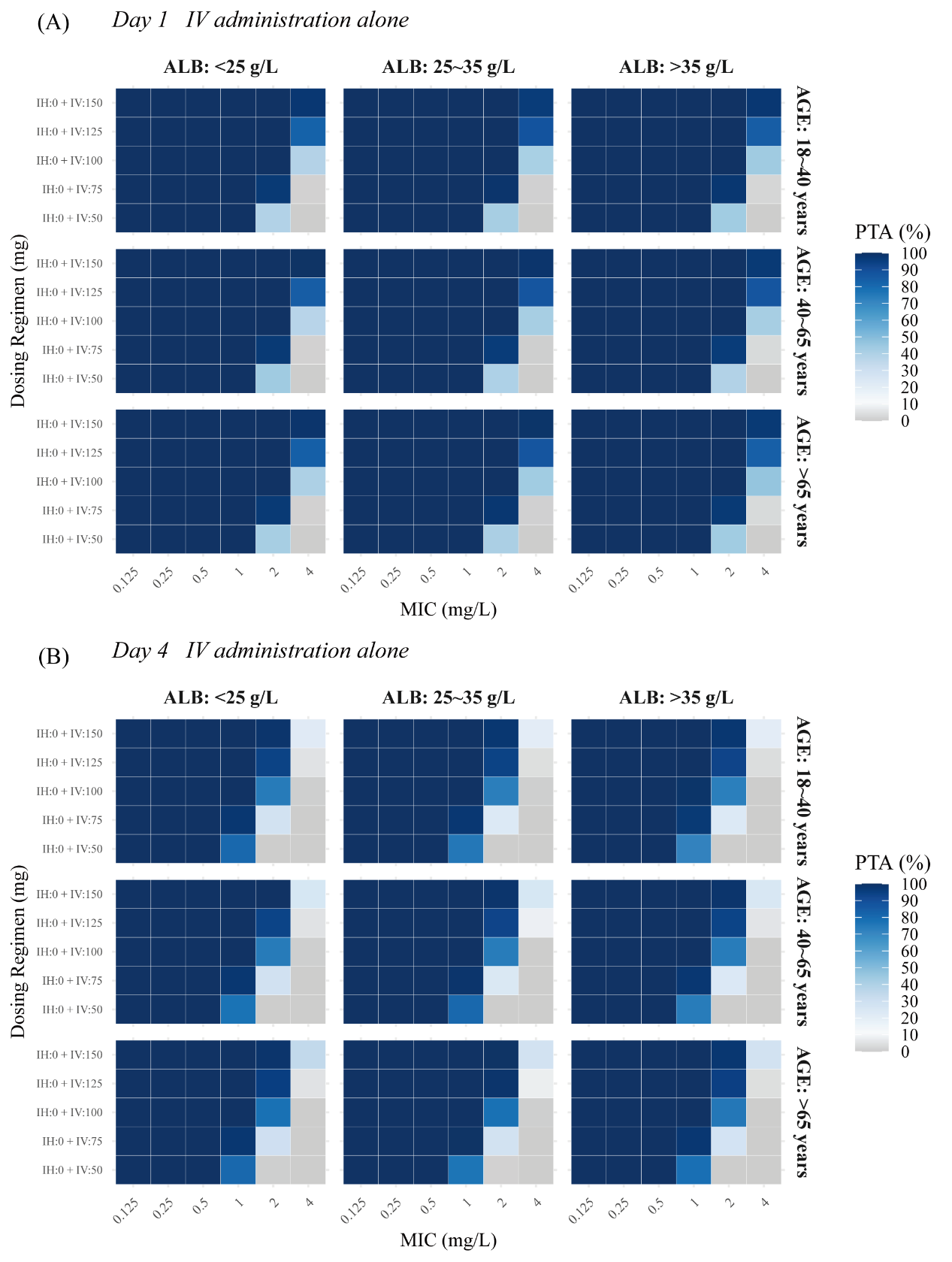


Figure S2. Probability of target attainment (PTA) in plasma at day 1 and day 4 (steady state) for dosage regimens (intravenous administration alone) across different ages and ALB levels in the final PopPK model. IH, nebulization maintenance dose; IV, intravenous maintenance dose.


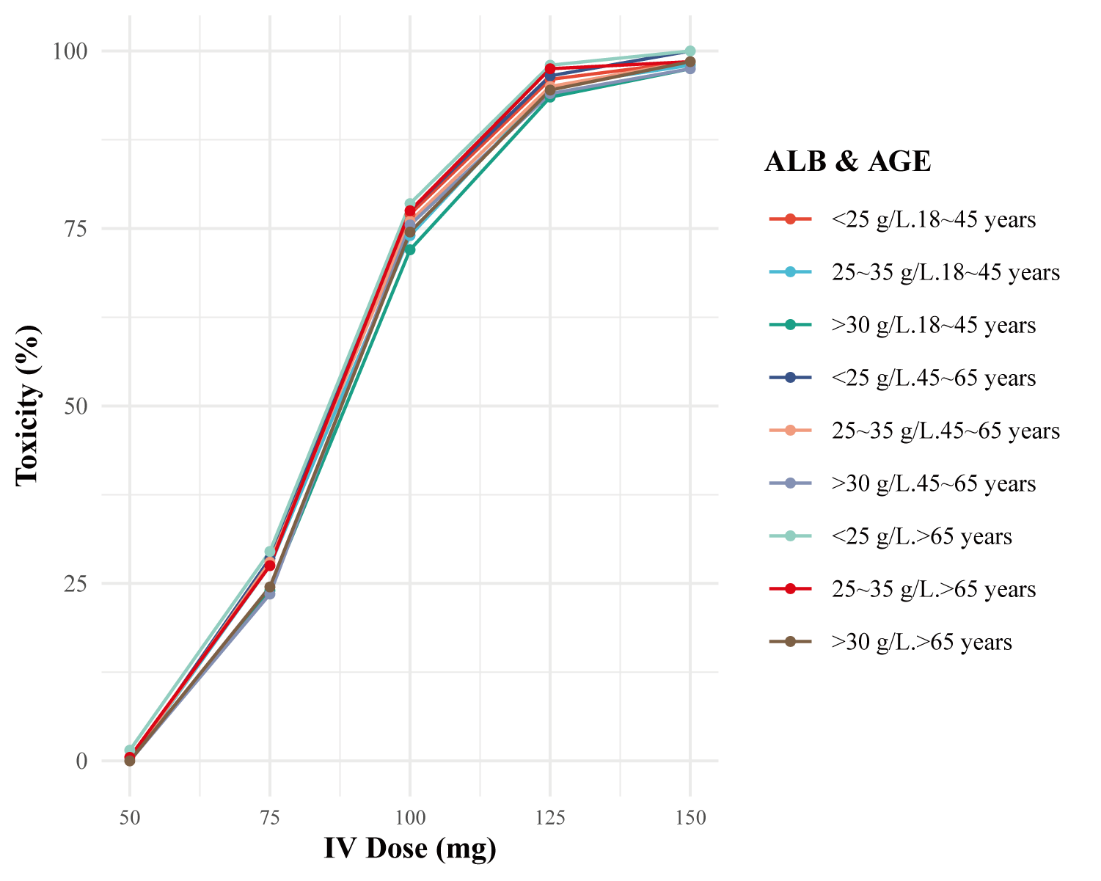


Figure S3. Incidence of toxicity (AUC_plasma_ > 100 mg·h/L) for different AGEs and ALBs at varying intravenous dosages.
