# Supplementary material for: Population pharmacokinetics study on nebulized and intravenous administration of polymyxin B in patients with pneumonia caused by multidrug-resistant gram-negative bacteria
Source: Antimicrob Agents Chemother. 2025 Apr 16;69(5):e00044-25. doi: 10.1128/aac.00044-25 (PMC12057357; doi:10.1128/aac.00044-25)
Supplement: Supplemental methods — Text and table. [file aac.00044-25-s0002.docx]

***Urea Assay in Plasma and BAL (Urease-Glutamate Dehydrogenase Method)***

Urea concentrations in plasma and bronchoalveolar lavage (BAL) fluid were quantified using a standardized enzymatic urease-glutamate dehydrogenase (GLDH) assay. Urea is hydrolyzed by urease to produce ammonia, which reacts with α-ketoglutarate and NADH in the presence of GLDH, forming glutamate and NAD⁺. The rate of NADH oxidation, measured as absorbance decay at 340 nm (ΔA/min), correlates with urea concentration. Assays were performed on semi-automated biochemical analyzers (Chemray 800, Rayto Life and Analytical Sciences Co., Ltd., Shenzhen, China) at 37°C. Calibration utilized calibrator serum, with results calculated as:

$$\mathrm{Urea}\left( \mathrm{mmol}/L \right)= \frac{{\Delta A}_{\mathrm{sample}}\times C_{\mathrm{calibrator}} (mmol/L)}{{\Delta A}_{\mathrm{calibratorUrea}}}$$

Urea nitrogen (mg/dL) was derived by multiplying urea (mmol/L) by 2.8. Plasma samples were collected in EDTA/heparin tubes, and BAL fluid was centrifuged (3000×g, 10 min) to remove debris. All samples were stored at 2–8°C to prevent urea degradation. Sensitivity, accuracy, and precision were validated for both matrices. Samples below the limit of quantification (BLQ) were documented.

***Reagents and Instrumentation:***

Urea Enzymatic Assay Kit (Catalog Number: S03036, Batch Number: 20230614) manufactured by Rayto Life and Analytical Sciences Co., Ltd., Shenzhen, China.

***Reagents:***

- R1: Tris buffer (100 mmol/L), ADP (2.0 mmol/L), α-ketoglutarate (7.0 mmol/L), NADH (0.3 mmol/L), GLDH (0.5 KU/L).
- R2: Tris buffer (100 mmol/L), urease (5.5 KU/L).
  *Storage:* Reagents were stored at 2–8°C; post-opening stability was 1 month.

***Sample Preparation:***

- Plasma: Collected in EDTA/heparin tubes, centrifuged (3000×g, 10 min), and analyzed within 24 hours.
- BAL: Centrifuged (3000×g, 10 min) to remove cellular debris. Supernatant was aliquoted and stored at −80°C if not analyzed immediately. Hemolysis or turbidity prompted filtration (0.22 µm).

***Instrumentation:***
semi-automated biochemical analyzers (Chemray 800, Rayto Life and Analytical Sciences Co.,Ltd., Shenzhen, China ) with a 340 nm wavelength, 1.0 cm light path, and 37°C temperature control.

***Procedure:***

- Sample Preparation: Plasma samples (EDTA/heparin anticoagulated) were centrifuged at 3000×g for 10 min to remove cellular components. Hemolyzed samples were excluded.
- Assay Setup:

1. Plasma: Followed the standard single- or dual-reagent protocol (3.0 mL reagent + 0.03 mL sample).
2. BAL: Used the dual-reagent protocol (2.4 mL R1 + 0.03 mL BAL, 300 sec incubation, 0.6 mL R2) to minimize matrix interference. Absorbance readings (A₁, A₂) were taken at 30 sec and 150 sec.

*Validation:*

**Table: The Accuracy and Precision Metrics for Urea Assay**

| Sample Type | Linear range | Intra-day accuracy | inter-day accuracy | Coefficient of Variation |
| --- | --- | --- | --- | --- |
| Plasam | 0.5–100 mg/dL | 96.4%~103.7% | 95.2% ~ 105.4% | 3.7% ~ 10.3% |
| BAL | 0.5–100 mg/dL | 94.9%~104.8% | 91.3% ~106.9% | 4.3% ~ 13.2% |
